# A critical review of definitions of rural areas in Indonesia and implications for health workforce policy and research

**DOI:** 10.1186/s12961-022-00847-w

**Published:** 2022-04-27

**Authors:** Likke Prawidya Putri, Deborah Jane Russell, Belinda Gabrielle O’Sullivan, Andreasta Meliala, Rebecca Kippen

**Affiliations:** 1 Faculty of Medicine, Public Health, and Nursing, Universitas Gadjah Mada, Yogyakarta, Indonesia; 2 School of Rural Health, Faculty of Medicine, Nursing, and Health Sciences, Monash University, Bendigo, VIC Australia; 3 Menzies School of Health Research, Alice Springs, NT Australia; 4 Rural Clinical School, University of Queensland, Toowoomba, QLD Australia

**Keywords:** Rural definition, Rural health services, Health policy, Equity, Health human resources

## Abstract

**Background:**

Choosing the appropriate definition of rural area is critical to ensuring health resources are carefully targeted to support the communities needing them most. This study aimed at reviewing various definitions and demonstrating how the application of different rural area definitions implies geographic doctor distribution to inform the development of a more fit-for-purpose rural area definition for health workforce research and policies.

**Methods:**

We reviewed policy documents and literature to identify the rural area definitions in Indonesian health research and policies. First, we used the health policy triangle to critically summarize the contexts, contents, actors and process of developing the rural area definitions. Then, we compared each definition’s strengths and weaknesses according to the norms of appropriate rural area definitions (i.e. explicit, meaningful, replicable, quantifiable and objective, derived from high-quality data and not frequently changed; had on-the-ground validity and clear boundaries). Finally, we validated the application of each definition to describe geographic distribution of doctors by estimating doctor-to-population ratios and the Theil-L decomposition indices using each definition as the unit of analysis.

**Results:**

Three definitions were identified, all applied at different levels of geographic areas: “urban/rural” villages (Central Bureau of Statistics [CBS] definition), “remote/non-remote” health facilities (Ministry of Health [MoH] definition) and “less/more developed” districts (presidential/regulated definition). The CBS and presidential definitions are objective and derived from nationwide standardized calculations on high-quality data, whereas the MoH definition is more subjective, as it allows local government to self-nominate the facilities to be classified as remote. The CBS and presidential definition criteria considered key population determinants for doctor availability, such as population density and economic capacity, as well as geographic accessibility. Analysis of national doctor data showed that remote, less developed and rural areas (according to the respective definitions) had lower doctor-to-population ratios than their counterparts. In all definitions, the Theil-L-within ranged from 76 to 98%, indicating that inequality of doctor density between these districts was attributed mainly to within-group rather than between-group differences. Between 2011 and 2018, Theil-L-within decreased when calculated using the MoH and presidential definitions, but increased when the CBS definition was used.

**Conclusion:**

Comparing the content of off-the-shelf rural area definitions critically and how the distribution of health resource differs when analysed using different definitions is invaluable to inform the development of fit-for-purpose rural area definitions for future health policy.

**Supplementary Information:**

The online version contains supplementary material available at 10.1186/s12961-022-00847-w.

## Background

Redressing the urban/rural disparity in health outcomes is one of the fundamental challenges to achieving health equity. Although rural populations may not always have poorer outcomes compared to urban dwellers, lack of access to healthcare in rural areas—related to fewer healthcare workers and facilities—contributes to higher mortality and morbidity among rural dwellers than otherwise may occur [1–3]. Given this, implementing strategies to ensure an adequate supply of rural health workers with appropriate skills could improve rural population health overall. However, the deployment of health workers to rural communities where need is the greatest could depend on how rural areas are defined and applied in government policies.

Rural area definitions generally refer to classifications based on topography, access or distance to urban facilities, agricultural landscape or population density. These attributes are widely considered to characterize “rural” in research and policy discourse [4–6]. There is wide variability in how rural areas are defined in health policy and research between and within countries. Studies have referred to “rural” areas based on agricultural land use, population density, distance or travel times from urban centres, the extent of geographic isolation, or having a country-like environment [4, 7]. Some research in low- and middle-income countries (LMICs) also defines rural areas based on their reliance on primary-level healthcare facilities [8].

While different countries should define rural areas in ways that suit their local context, it is crucial that within countries, some consensus is reached about what is and is not a rural area [4–7]. This is particularly important within a specific field of inquiry, such as the health workforce, so that there is consistency at a national level. The absence of standardized rural area definitions for health purposes within countries poses a major challenge for effectively targeting rural health policy and programmes and supporting comparative research [5, 6]. The potential impact is likely to be greater in LMICs, where relatively more people live in rural areas and may be substantially more disadvantaged unless supported by rural-targeted health interventions [9]. Moreover, there is a limited rural investment in the low-resource environments of many LMICs; hence, it is crucial to ensure that scarce resources are targeted to precisely defined rural areas where need (and impact) is likely to be greatest [9, 10].

Indonesia is an LMIC with a large rural population and persistent inequality in accessing healthcare services and health outcomes between its regions [11–14]. In Indonesian-based studies on health workforce, the term “rural” is often inconsistently defined and relies on subjective perception [7, 8]. Some refer to rural as any location outside Java-Bali, the most developed areas in the nation [15, 16], whereas others classified areas as rural based on population size or according to researchers’ or respondents’ opinions [17–19]. On the other hand, a range of studies about healthcare utilization and outcomes, which analysed the national surveys [11, 14], classified Indonesian areas according to the Central Bureau of Statistics (CBS) definition of urban and rural villages [20, 21]. The latter definition was different from the definitions used in the health workforce studies. From a health policy perspective, there are currently various definitions of a rural area applied in policies on providing scholarships for specialist training, deploying doctors in financially incentivized rural posts, and allocating additional incentives that could have implications for health workforce distribution [22–24]. Differences in the definition of rural among studies and policies could prevent the uptake of research evidence into practice. Likewise, policies that are not guided by evidence could be less effective in achieving their purposes [25].

Given this background and to formulate a more suitable rural area classification for health policy and research in Indonesia, this study aimed at exploring and reviewing the existing definitions of rural areas in Indonesian health policy and research, particularly in the health workforce. This aim is achieved by (1) summarizing the context, actors and process of establishing the existing rural definitions, (2) comparing the content of the definition through assessing their advantages and disadvantages and (3) further statistical exercise to validate these definitions by applying them to Indonesian medical workforce distribution data. The study results could help in developing and reaching consensus on a rural area classification that fits for the health policy and research.

## Methods

### Design

This study undertook qualitative document analysis of the “off-the-shelf” rural area definitions used in Indonesian health policies and quantitative validation of the definitions using Indonesian medical workforce data.

### Data collection

Rural area definitions were identified by searching policy documents publicly available through official Indonesian government websites and other relevant websites, including the National Legal Documentation and Information Network (NLDIN), Ministry of Health (MoH), Ministry of Villages, Development of Disadvantaged Regions, and Transmigration (MoVDT), CBS and the World Bank open repository. The key search terms in Indonesian were “urban” *(kota* or *perkotaan*), “rural” (*desa* or *perdesaan*), “remote” (*terpencil*), “less developed” or “underdeveloped” or “disadvantaged” (*tertinggal*). Documents were also sourced peer-reviewed articles on health workforces from selected databases (Medline, EMBASE, Google Scholar) using keywords ”doctor”, “physician” or “health workforce”, “rural” or “remote” or “underdeveloped”, and “Indonesia”. Initial searches were conducted by LP. Documents were added that were sourced from key Indonesian health stakeholder contacts that were known to the two authors (LP and AM). All authors developed the inclusion criteria: (1) the full-text article or document was available in either English or Indonesian; (2) published between 2000 and 2020; (3) included a definition of “rural” or “remote” or a particular area classified geographically; (4) related to the health sector, and, for peer-reviewed articles, (5) original research, policy or literature review. Because the policy documents were in Indonesian language, these were screened by LP and AM, who are native speakers of Indonesian language, to assess whether they met the previously stated inclusion criteria.

Additional criteria specifically for rural area definitions included (1) that they were applied to health-related policy or programmes AND (b) they used an explicit scoring system to define criteria. The initial screening and assessment of rural area definitions was also completed by LP and AM and discussed with all authors. All authors agreed on the final rural area definitions included in this study.

### Data analyses

The data analyses were completed in three iterative phases.

*Phase 1* included the review of the context, actors and the processes for a range of rural area definitions applied in Indonesian health policy. For each rural area definition meeting the inclusion criteria, a deductive approach was used to summarize relevant policy actors, processes, and context of each definition drawing on Walt and Gilson's policy analysis triangle [26]. The aim of exploring these aspects was to help inform the potential impact of the rural area definitions on policies around current and future health workforce deployment.

*Phase 2* assessed the contents of each definition by reviewing its advantages and disadvantages guided by the norms of appropriate rural definitions for health policy and research formulated by Hart et al*.* (2005) and Coburn et al. (2007) as to whether each definition was explicit and meaningful; replicable; quantifiable and not subjective; derived from high-quality data; not frequently changed; had on-the-ground validity; and had clear boundaries [5, 6]. As these references did not include a detailed description of each norm, the authors discussed how each norm was relevant to the rural health workforce.

*Phase 3* compared the doctor-to-population ratios (DPRs) and inequality estimates between districts when grouped according to the identified rural area definitions. This was done to further inform the validity of each definition in pinpointing areas with lower doctor supply.

First, to ensure comparability, all definition were converted to the district level. District level was selected because Indonesian governance is decentralized to the district level; thus, analysis was expected to inform district health policies and programmes. The area of districts ranges from 10 to 44,071 km^2^, whilst population size in 2018 ranged from 13,800 to 5,840,000 [27]. Each district is administratively divided into subdistricts, and subdistricts comprise several villages.

District-level DPRs were calculated as the number of medical doctors (of any type) in each district, divided by the district's population size. Data used for DPR calculations reported (1) the number of doctors residing in each village in 2011, 2014 and 2018, sourced from the village censuses (*Potensi Desal*, PODES), and (2) district-level population sizes in 2011, 2014 and 2018, sourced from the CBS [27–29]. Village censuses are conducted every 3–4 years. They collect information on population characteristics, road infrastructure, and health and educational facilities, with village officials as the informants. The district-level population size was derived from annual population estimates based on updates to the 2010 Indonesian Population Census count, available from provincial CBS websites. Population censuses are conducted every 10 years.

Quantitative analyses included descriptive statistics and decomposition of Theil-L based on the DPRs. DPRs are a key indicator for monitoring doctor supply relative to population need [30] and informing achievement of the Sustainable Development Goals between countries [31]. In addition, studies suggest that DPRs are valuable to identify inequalities in the distribution of doctors within countries [32, 33].

The Theil-L measures—Theil-L total and its within-group (*L*_*W*_) and between-group (*L*_*B*_) decompositions—are frequently applied to DPRs to investigate inequalities in health workforce distribution and the sources of these inequalities [34]. The Theil-L (L) ranges between 0 and 1, with higher values indicating higher inequality. Ideally, each category of a rural area definition is relatively homogeneous (within-group variance accounts for a small proportion of Theil-L), while the categories themselves are heterogeneous (between-group variance accounts for a large proportion of Theil-L). In this study, the decomposition of the Theil-L estimates for each category of the rural area definitions allows us to identify whether the between- or within-group differences are more responsible for overall DPR inequality.

All analyses were completed using StataIC 16.0 (StataCorp, College Station, TX, USA).

Box 1. Theil-L estimates Theil-L (L) formula:

$$L=\sum i \left(\frac{{p}_{i}}{P}\right)[\mathrm{log}\left(\frac{{p}_{i}}{P}\right)-\mathrm{log}(\frac{{d}_{i}}{D})]$$

${p}_{i}$ = population size at unit i, *P* = overall population size, ${d}_{i}$ = number of doctors at unit i, *D* = overall number of doctors.

$$L = L_{w} + L_{B}$$, with:

$$L_{W} = \, \left( {P1/P} \right)L1 + \, \left( {P2/P} \right)L2$$

$$L_{B} = \, \left( {P1/P} \right) \, \log \, \left( {X/X1} \right) \, + \, \left( {P2/P} \right) \, \log \, \left( {X/X2} \right)$$

*P* = overall population size, *P1* = population size in group 1, *P2* = population size in group 2, *L1* = L measure in group 1, *X* = overall doctor density, *X1* = doctor density in group 1, *X2* = doctor density in group 2, *L2* = L measure in group 2.

## Results

**Fig. 1:**
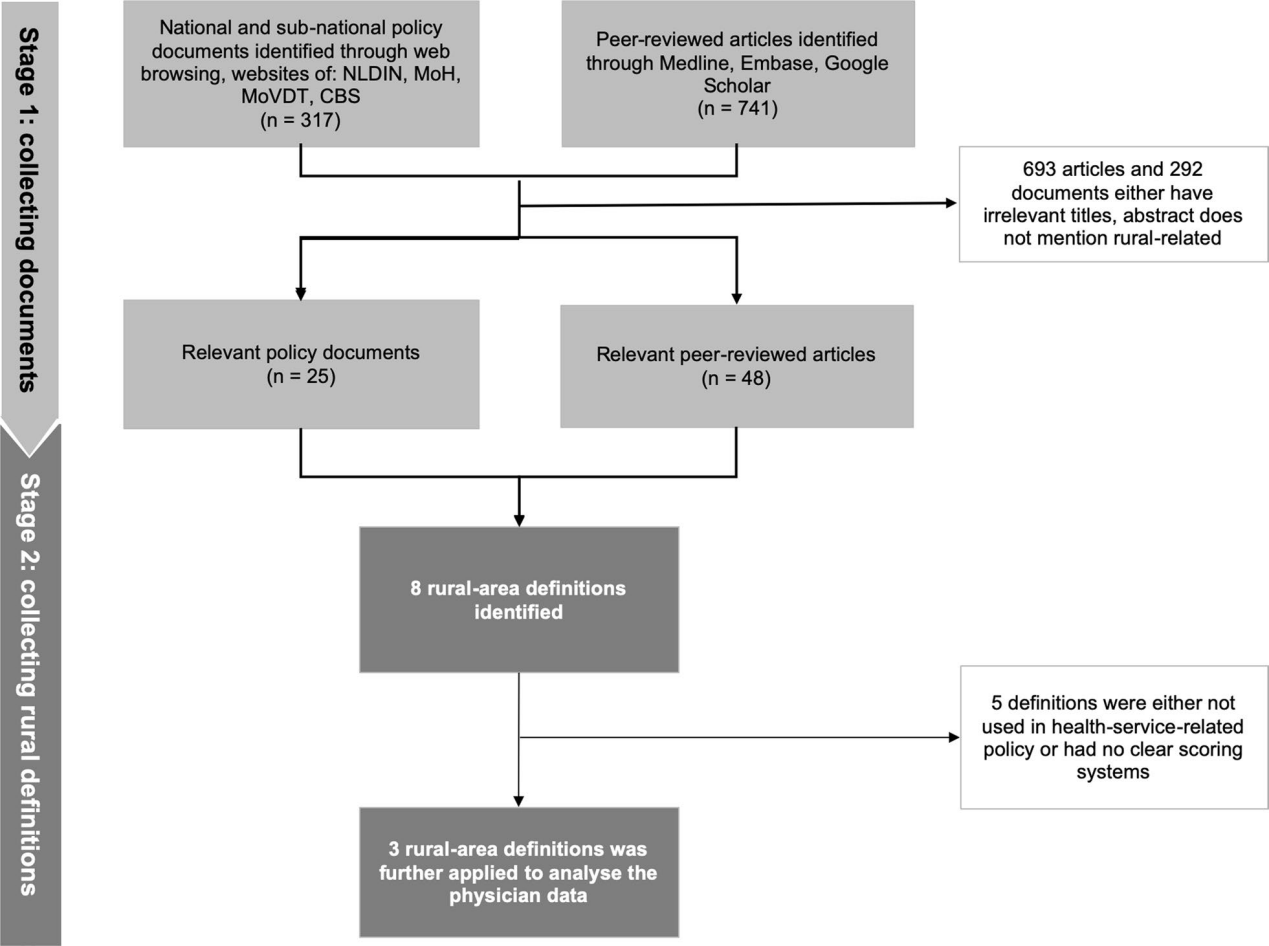
Search strategy to identify rural area definition. MoH: Ministry of Health (Kemenkes), MoVDT: Ministry of Villages, Development of Disadvantaged Regions, and Transmigration (Kemendesa PDTT), NLDIN: National Legal Documentation and Information Network (JDIH), CBS: Central Bureau of Statistics

The search identified 25 policy documents (Additional file 1: Table 1A) and 48 articles containing eight rural area definitions. Of these, only three definitions were applied in health-related policy or programmes and had a clear scoring system, thereby meeting the inclusion criteria. Figure 1 shows the flow for the documents and rural definition search. The three definitions were as follows: (1) MoH-defined remote health facilities, (2) presidential regulation-defined less developed districts and (3) CBS-defined urban and rural villages (hereafter, these are referred to as MoH definition, presidential definition and CBS definition, respectively). Definitions that were excluded are (1) district nomenclature (*Kota/Kabupaten*), (2) underdeveloped, border, island areas (DTPK: *Daerah Tertinggal, Perbatasan, dan Kepulauan),* underdeveloped, frontiers, outermost areas (D*aerah* 3T: *Tertinggal, Terdepan, Terluar*), village nomenclature (*Desa*/*Kelurahan*) and regional (Sumatera, Java-Bali, Kalimantan, Sulawesi, Nusa Tenggara-Maluku-Papua). The explanations on the inclusion or exclusion are available in the Additional file 1: Table 2A.

The three rural area definitions apply at different geographic levels. The CBS definition is at the village level, the smallest compared to the other two definitions. The MoH definition applies to health-facility catchment areas, which may include one or more villages, while the presidential definition is determined at the much larger district level. In 2018, there were 2054 remote health facilities, 122 less developed districts and 67,602 rural villages, according to the MoH, presidential and CBS definitions, respectively. Some overlaps were identified across the three definitions (39% of the remote facilities were located in rural villages in less developed districts) or two definitions (i.e. 53% of remote facilities were in rural villages in more developed districts, and 2% were in urban villages in less developed districts). The remaining 6% of remote facilities were in urban villages located in more developed districts (Table 1).

**Table 1:** Remote health facility^1^ locations according to the presidential and CBS definitions

CBS definition^2^	Presidential definition^3^		Total
	More developed district	Less developed district	
Urban villages	144	51	195
Rural villages	1325	984	2309
Subtotal	1469	1035	2504

Source of data: MoH, 2018

^1^According to MoH letter DG.01.01/II/1979/2018. All of the remote health facilities were owned by the government (*Puskesmas*)

^2^According to the Head of CBS Regulation 37/2010, 67,602 villages were classified as rural and 16,329 urban in 2018

^3^According to Presidential Regulation 131/2015, 122 districts are considered less developed and 392 as more developed in 2018

**Fig. 2:**
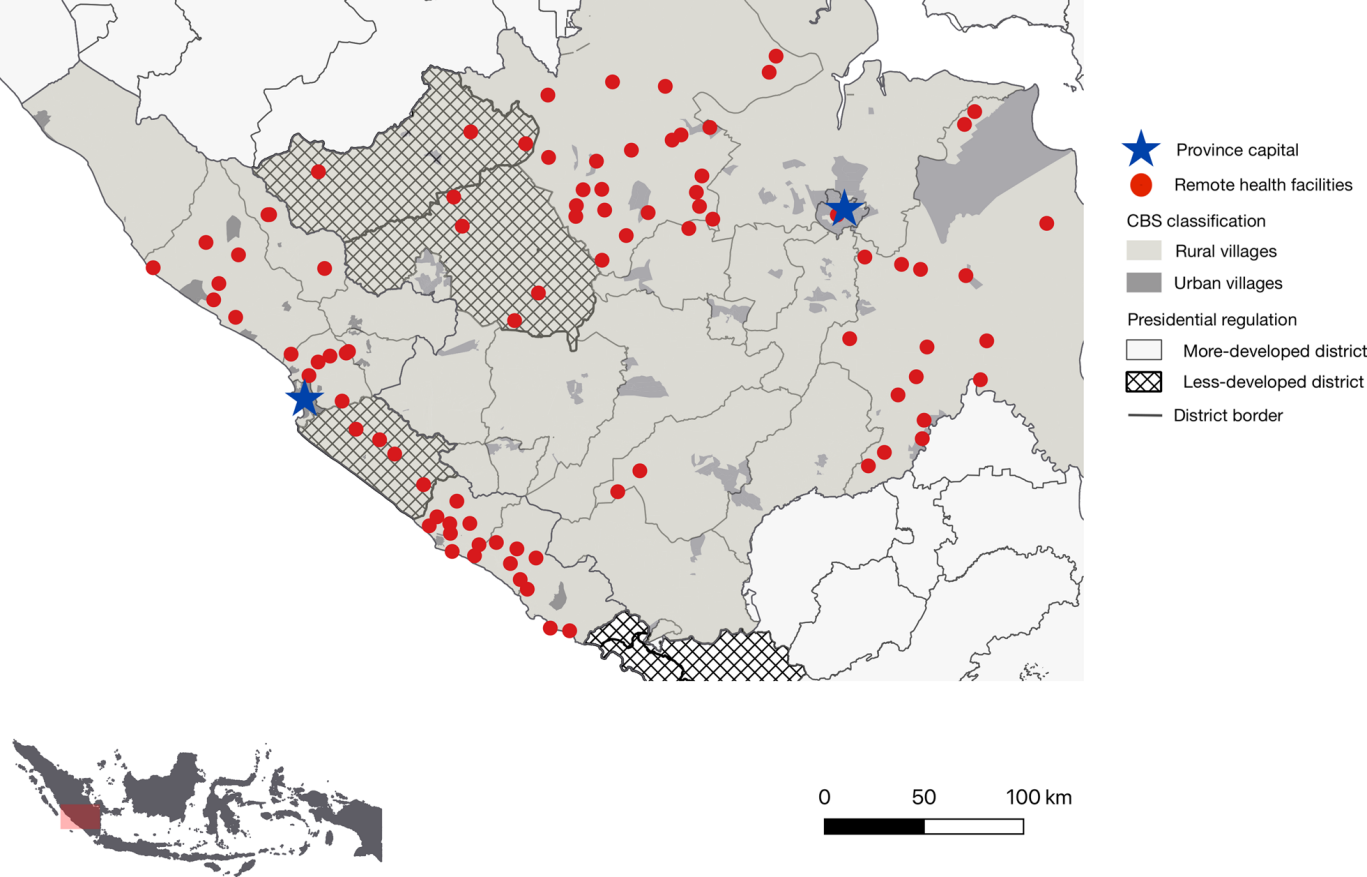
Map of remote health facilities, less/more developed districts, and urban/rural villages in the provinces of Bengkulu and South Sumatera. Illustrates the locations of the three rural area definitions in Bengkulu and South Sumatera—provinces located in Sumatera Island. The proportion of less developed districts and rural villages varies across provinces. For example, 10% of districts in Bengkulu and 12% in South Sumatra are less developed, compared to 90% in Papua and 0% in Central Java. And 89–90% of villages in Bengkulu and South Sumatera are rural, compared to 97% and 67% in Papua and Central Java (Additional file 1: Table 3A)

Figure 2 illustrates the locations of remote health facilities (MoH-defined), less/more developed districts (presidential regulation-defined), and urban/rural villages (CBS-defined) in two selected provinces in Indonesia and how these definitions may overlap.

### Phase 1: actors, processes, contexts and contents of the rural area definitions

The CBS definition was the first urban/rural classification established by the government, regularly updated every 10 years since 1971 [35]. The less developed district classification was defined through presidential regulations since 2004, updated every 5 years [36], while the remote health facility definition was established by the MoH initially in 2007 with occasional updates [37]. The CBS definition was developed to promote more uniform urban/rural concepts throughout Indonesian policy and strategy [35], while the MoH and presidential definitions were released in conjunction with the national strategy to accelerate the growth of less developed and more remote Indonesian areas [38]. This national strategy was triggered by the newly elected government’s commitment to equitable development across Indonesia, by supporting the growth in the eastern region which had been experiencing long-term below-national-average economic, educational and health outcomes [39, 40]. In 2004, the commitment was strengthened to include any underdeveloped Indonesian regions—whether located in the eastern region or not—highlighting the importance of accelerating development in the less developed and remote areas. Following this, several ministries established policies to achieve better equity between regions, including the MoH, which began classifying remote health facilities in 2007 (Table 2).

**Table 2:** Actor, process, context and content of the rural area definitions

	MoH definition	Presidential definition	CBS definition
Actors (who established the definition)	Established by the MoH	Established by the president, with more detailed technical guidelines issued by the MoVDT	Established by the CBS
Process (when the definition was established)	One of the MoH responses to the Indonesian Government’s National Long-Term Development Plan 2005–2025, to accelerate the growth of less developed and more remote Indonesian areas. Regulations for remote health facilities have been established or revised three times, in 2007, 2013 and 2015	In conjunction with the National Long-Term Development Plan 2005–2025 to accelerate the growth of less developed and more remote Indonesian areas. The definition was updated in 2010, 2015 and 2020	The latest urban/rural classification was released in 2010, as the update from previous versions (1971, 1981 and 1990), using the 10-yearly population census data
Context and content (purpose of the definition and use in the health service policy)	The definition was aimed to improve healthcare access and quality in remote and very remote areas, strengthen community empowerment and provide legal certainty for healthcare workers Guide for deploying health workers under the rural financial programme (i.e. *Nusantara Sehat*, voluntary contractual posting or *PTT Daerah*) A higher capitation rate is allocated for remote health facilities, of which at least 50% must be given for health personnel incentives. Remote health facilities receive a capitation payment rate at least twice as much as their non-remote counterparts (i.e. non-remote facilities with one full-time doctor will receive IDR 4500, while remote facilities with one full-time doctor will receive IDR 10,000). Remote facilities can also receive a capitation fund for 1000 members even if the actual number of members is lower	The definition aimed to accelerate the reduction of the gap between regions to achieve more equitable development and supply the basic needs, facilities and infrastructure in the less developed areas Doctors working in less developed districts are prioritized for scholarships and recommendation letters from the local government for specialist education Health facilities, both primary healthcare centres and hospitals, are prioritized for special funding for building healthcare infrastructure. The quality of health facilities is important to recruit and retain health workers in rural and remote areas	The classification was aimed to promote uniformity in the use of concepts, definitions and criteria for urban and rural areas in Indonesia The MoH classifies government-owned primary healthcare facilities (*Puskesmas*) as urban or rural based on a modified CBS classification. However, neither the original nor modified CBS classification has been used in health-funding policy; there is no substantial difference between urban and rural *Puskesmas* regarding the scope of services and capitation rate received

*MoH* Ministry of Health, *MoVDT* Ministry of Villages, Development of Disadvantaged Regions, and Transmigration, *CBS* Central Bureau of Statistics

These definitions have different impact to health workforce geographic distribution (see context and content in Table 2). The MoH definition determines that the capitation payment rate for the remote health facilities is at least twice that of their non-remote counterparts [41], resulting in higher incentives that could be received by doctors practicing in those facilities. The MoH-defined remote facilities also can employ doctors under the financially incentivized contractual posting such as in *Nusantara Sehat* [22]*.* Doctors working in the presidential-defined less developed districts are prioritized to obtain scholarships for specialist trainings [24]. In addition to these, doctors working in remote health facilities or less developed districts are entitled to receive additional financial and nonfinancial benefits such as hardship allowances and government-provided accommodation. Further, facilities classified as remote or situated in less developed districts are more likely to receive special funding to build health infrastructure [42]. All of these policies could potentially encourage more doctors to work in these rural-defined places (i.e. the less developed districts and remote health facilities). On the other hand, while the CBS definition was applied to classify government-owned primary healthcare clinics (*Pusat Kesehatan Masyarakat* [*Puskesmas*]) into urban and rural, no special funding or financial incentives were provided due to this classification received [43]. The summary of contexts and contents can be found in Table 2.

### Phase 2: advantages and disadvantages of each rural area definition

Table 3 shows the advantages and disadvantages of each definition to serve as the appropriate rural area definition for health research and policy purposes.

**Table 3:** Summary of strengths and weaknesses of rural area definitions

Norm	Description	MoH definition	Presidential definition	CBS definition
Explicit	Taxonomy criteria are clear	Strengths: The definition has a clear set of criteria and scoring systems Based on 12 criteria, with a score range of 0 to 12, those scored 3+ are classified as remote health facilities Criteria include topography (e.g. mountain, coast, inland, small island), transportation and access to the nearest district centre, susceptibility to conflict or natural disaster (volcano eruption, earthquake, landslide), security conditions and access to food-related staples	Strengths: The definition has a clear set of criteria and scoring systems Based on 27 criteria, each weighted 1.43–10%. The higher the score, the less developed the district. The top 60% of districts are classified as less developed Criteria include economic development indicators (per capita consumption, proportion of people living in poverty); local fiscal capacity; human development measures (life expectancy, population literacy, length of schooling); geographic accessibility (distance to the district centre and health and education facilities; health facilities, doctors, schools per 1000 population); road infrastructure; and vulnerabilities to natural disaster or conflict	Strengths: The definition has a clear set of criteria and scoring systems Based on eight criteria, with a score range of 2–22. Villages with a score of less than 10 are classified as rural Criteria comprise population density; agricultural-based household income; availability of schooling, health and business facilities; and access to electricity and a phone landline
Meaningful	Has at least one criterion that is associated with higher doctor density in a previous study	Weaknesses: It does not account for population characteristics that are associated with doctor supply	Strengths: Accounts for social determinants of health (e.g. literacy and per capita income), associated with higher doctor supply in other nations	Strengths: Accounts for population characteristics (e.g. population density) strongly associated with doctor supply in other nations
Replicable	The taxonomy can be applied at another level of government or can be applied again in the future	Weaknesses: The classification is at the facility level with no clear geographic boundary. Hence, it would be difficult to apply to another level. However, it may be possible to reclassify districts with these characteristics	Weaknesses: The classification is at the district level; hence, it cannot be applied at the subdistrict or village level	Strengths: Yes, the classification is at the village level and available to be reclassified at the subdistrict, district or provincial level
Derived from available, high-quality data	The taxonomy is based on high-quality, accessible data	Weaknesses: It relies on local and national government’s information on the selected criteria, thus was not derived from available high-quality data	Strengths: Derived from population census, national survey and fiscal data	Strengths: Derived from population census data
Quantifiable and not subjective	The taxonomy is based on objective measurement	Weaknesses: Provincial and district health authorities can nominate facilities that meet remoteness criteria, and the MoH then reviews the nominations prior to approval. Therefore, this process could be subjective based on local area knowledge/justification of facility remoteness, thus may overlook health facilities located in a district with less proactive provincial or district health authorities	Strengths: Scores are calculated by the national government based on objective and thorough assessment of demographic, economic, infrastructure and access, thus objective, with formal national status, aiding interpretation	Strengths: Scores are calculated by the national government based on objective and thorough assessment of demographic, economic, infrastructure and access, thus objective, with formal national status, aiding interpretation
Has on-the-ground validity	The taxonomy is valid in demonstrating the rurality or remoteness of a selected area	Strengths: Based on local area knowledge/justification of facility remoteness, thus offers a better nuance in describing the area’s limited geographic accessibility	Weaknesses: It is measured at a larger geographical (district) level, potentially missing nuances of geographically isolated villages and population access The composite index produced by the criteria has a wide range, but the final classification only groups areas into two categories: less/more developed districts	Strengths: Measured at a smaller geographical (village) level, thus could have greater validity in describing geographic and accessibility conditions in a localized area
				Weaknesses: There is high weighting proportion for population density for this scoring. For example, areas with high population density (> 8500/km^2^ get 8 points, with a hairdresser available get 1 point and household with electricity ≥ 90% get 1 point, making a total of 10 points, hence classified as urban. Meanwhile, such a place, when having no hospital within 5 km or primary health facilities with no doctors, might have been classified as urban. Therefore, there might be several urban villages with poor public and health facilities, thus overlooked when government needs to support areas with such characteristics. Any increased resources would be spread thinly between a large number of rural villages The scoring ranges from 2 to 22, but the final classification only groups areas into two categories: urban/rural villages
Boundary	Has a clear area boundary, either geographical or political	Weaknesses: Each facility covers one or more villages, making the area boundary unclear	Strengths: The boundaries, both geographical and governmental (political), between districts are clear	Weaknesses: The boundary between villages is unclear. And a doctor may live in one village while working in others, thus distorting the actual supply of doctors
Update frequency	Data used for taxonomy are updated periodically	Weaknesses: The MoH update the list of remote facilities yearly; however, the classification of a remote facility can be done any time based on request approval	Strengths: It is regularly updated (every 5 years) using the latest census or survey (linking it with population and infrastructure development)	Strengths: It is regularly updated (every 10 years) with the latest population census (linking it with population and infrastructure development)

All three definitions are explicit in terms of having clear criteria measuring the distance or accessibility of urban amenities such as district business centre, health and education facilities (Table 3).

The presidential and CBS definitions use criteria meaningful for medical workforce supply by accounting for population characteristics positively associated with higher doctor need and/or utilization including population density, per capita consumption, and literacy**.** On the other hand, the MoH remote health facility classification does not account for population density.

While the presidential and CBS definitions systematically classify areas based on high-quality, routinely collected data, minimizing subjectivity and resulting in standardized outcomes, the MoH-defined remote facility definition allows some provincial and district health authorities to recommend health facilities for inclusion. The remote facility classification, being partially self-nominated by local government, offers enhanced flexibility and considers local challenges in supplying health resources that are not necessarily captured by existing criteria used in other definitions, enabling enhanced validity in demonstrating remoteness. However, this self-nominating procedure could also introduce subjectivity and possible bias—different districts could have varying interpretations of when a health facility is eligible to be classified as “remote”.

The presidential-regulated definition is applied at the district level, which has a clear area boundary and gives it the advantage of being pragmatic for informing policy since the governance is decentralized to the district level. However, the classification at the district level would not allow replication at the lower level of administrative governance (e.g. village level). Due to the large size of districts (relative to villages), this definition does not account for different geographic situations within a district, and thus has limitations in its ability to pinpoint significantly disadvantaged areas within a more developed district. Also, 30% of the scoring weight is based on economic development, and only 4% of the total scoring weight for this definition is based on healthcare access, which, for example, may disadvantage areas with poor health access but relatively high fiscal capacity.

The CBS definition applies at the village level and offers greater detail in demonstrating rurality; however, the boundaries between villages are less clear than between districts. Further details on the scoring system are included in Additional file 1: Table 4A, 5A, and 6A.

### Phase 3: validating the identified rural area definitions using data on Indonesian doctors

The DPRs for the three definitions are shown in Table 4 (the three rural area definitions were adjusted at district level for the comparison purposes; Additional file 1: Table 7A). In general, DPRs were lower in the more rural districts, irrespective of which definition was used.

**Table 4:** The ratio of doctor per 100,000 population (DPR) at district level 2011–2018

Geographic classification	2011		2014		2018	
	DPR	Min, max	DPR	Min, max	DPR	Min, max
Indonesia	24	1, 668	23	2, 145	24	2, 181
MoH definition^1^						
District without remote health facilities	28	2, 668	24	3, 145	26	3, 181
District with remote health facilities	19	1, 191	20	2, 110	22	2, 105
Presidential definition^2^						
More developed district	26	3, 668	24	3, 145	25	4, 181
Less developed district	17	1, 134	18	2, 69	19	2, 88
CBS definition^3^						
Quintile 1 (most urban)	37	4, 159	39	9, 145	40	8, 181
Quintile 2	25	5, 326	21	6, 110	22	5, 104
Quintile 3	17	4, 60	18	4, 62	18	5, 57
Quintile 4	16	3, 45	17	3, 52	19	4, 59
Quintile 5 (most rural)	23	1, 667	17	2, 69	19	2, 88

Source: The doctor data was calculated from the number of doctors residing in each village according to Village Census 2011, 2014 and 2018. The number of populations was projected estimation in 2011, 2014 and 2018 according to Population Census 2010

^1^The list of remote health facilities was based on district head decree that was verified by MoH letter DG.01.01/II/1979/2018

^2^The classification of remote districts was based on Presidential Regulation 131/2015, where 122 districts are considered less developed and 392 as more developed in 2018

^3^The classification of the urban/rural village was based on the CBS Regulation 37/2010, where 67,602 villages were classified rural and 16,329 urban in 2018

The Theil-L decomposition (Table 5) shows that, for all three definitions, the inequality of doctor distribution in 2018 was mainly attributable to within-group differences (76–98%) in the categories used by the respective definitions. The contribution of between-group differences (*L*_B_) to the overall inequality was highest when districts were grouped according to the CBS definition. Between 2011 and 2018, the *L*_B_ of CBS definition increased from 14 to 22%, but that of the MoH and presidential definitions decreased from 5 to 2% and 4 to 3%, respectively.

**Table 5:** Inequality measures of the DPR according to the rural area definitions

Year	Theil-L total^1^	Theil-L decomposition					
		MoH definition^2^		Presidential definition^3^		CBS definition^4^	
		% Within group^5^	% Between group^6^	% Within group^5^	% Between group^6^	% Within group^5^	% Between group^6^
2011	0.33	95.14	4.86	96.00	4.00	85.75	14.25
2014	0.24	97.12	2.88	96.23	3.77	76.27	23.73
2018	0.22	98.38	1.62	96.86	3.14	78.34	21.66

Source of data: The data was obtained from the number of doctors residing in each village according to Village Census 2011, 2014 and 2018. The population size in the corresponding years was derived from the projection of Population Census 2010. The DPR was calculated at the district level

^1^Theil-L total of DPR in Indonesia

^2^Districts classified as with or without a remote health facility according to MoH letter DG.01.01/II/1979/2018

^3^Districts classified as less developed or others according to Presidential Regulation 131/2015

^4^Districts classified into five quintiles of the proportion of population residing in rural villages according to the CBS Regulation 37/2010. See Additional file 1: Table 7A for more detail information

^5^Decomposition of Theil-L that reflects the difference in DPR within each group (*L*_W_)

^6^Decomposition of Theil-L that reflects the difference in DPR between groups (*L*_B_)

For the inequality estimates, a perfect rural area classification would show 100% L-between-group and 0% L-within-group differences—the higher the L-between difference, the better ability to determine the source of inequality for each group. Of the three definitions, the CBS definition has the lowest L-within-group difference (76–86%), which means it was best at grouping areas with similar doctor density. Although the CBS definition performs better than other definitions, its Theil-L-between is still far higher than ideal.

## Discussion

This study is the first to identify and critically analyse the different rural area definitions applied in Indonesian health policy and validate the definitions by analysing empirical data on doctor distribution. No single definition fulfilled all the criteria for an appropriate urban/rural classification for health policy (i.e. meaningfulness, replicability, validity, objectivity, derived from high-quality data and has a clear area boundary). However, irrespective of which definition was used, each defined rural area was consistently associated with lower doctor density than its non-rural area counterpart. Among the strengths of presidential and CBS definitions were the following: being meaningful for medical workforce supply, as they captured important characteristics that are commonly correlated with doctor density (i.e. population density and per capita income), objective, quantifiable and derived from nationally available and high-quality data; hence, reliable to detect areas that are more rural or remote than the others. The strength of the MoH-defined facility definition was that it was partially self-nominated by the local government; hence it has an enhanced ability to identify areas with limited healthcare supply. However, it makes the MoH definition less objective than the other two and could have underestimated the actual number of remote facilities.

The Theil-L between-group value ranged from 2 to 24%, far below the ideal figure of 100%, suggesting that the doctor density inequality was primarily attributed to the different characteristics within the groups (which in this case are rural area definitions) rather than differences between groups. More advanced analysis is warranted to identify factors contributing to these within-group and between-group differences. Such analyses could strengthen existing evidence-based approaches in developing rural area definitions for use in health policy and research to improve health equity.

Based on our analyses of contexts and content of different definitions, the presidential (less developed district) and MoH (remote facility) have guided government investment in influencing geographic distribution of doctors. These definitions have been applied to determine which doctors are prioritized for specialist scholarships, and who is eligible for financial incentives (i.e. in the *Nusantara Sehat* programme). Both the scholarships and financial incentives are important factors associated with doctors working in rural and remote locations in LMICs [8]. In contrast, the CBS (urban/rural village) definition is not currently used in government health policies for attracting rural doctors. This is despite it having the highest Theil-L between-group value of the three definitions, which otherwise suggests that it may be better in differentiating areas with low DPRs. These findings suggest that adopting a rural area definition such as the CBS definition, which incorporates urban/rural village characteristics when targeting future health workforce policies, could better address the imbalanced doctor distribution in Indonesia.

Investigating predictors of doctors' geographic distribution and incorporating the identified predictors into the rural area definition could be an essential part of developing a more suitable classification of rural areas for use in health workforce policy. Humphreys et al. (2012) explored the factors associated with doctors’ work location in Australia—total hours worked, public hospital work, on-call after hours, difficulty taking time off, partner employment, and schooling opportunities—and how these varied across areas grouped by population size. This informed the development of the Modified Monash Model classification that guides the allocation of rural retention incentives to Australian primary care doctors [44]. Work of this nature requires collecting and accessing the appropriate medical workforce data at a national level. Future research in Indonesia could also involve using geographic information system (GIS) methods to assess the spatial accessibility of doctors and other health resources [45–47].

Besides the need for more advanced studies, many contextual aspects relevant to a country’s health system should be considered in determining a rural area definition for future policy and research. For example, in the Indonesian setting, the health governance—including the health workforce management—is decentralized at the district level. Developing rural area definitions measured at the district level could benefit decision-making more than if they were measured at a different level like subdistrict, a health facility’s catchment area, or village. Collaboration between the government, as decision-makers, and researchers is warranted to ensure that all important contexts are considered in developing a fit-for-purpose rural area definition for future health workforce policies.

While our study focused on critiquing rural area definitions, it is critical to note that the Theil-L total decreased over a 7-year period, suggesting an improved doctor distribution between districts. The differences in doctor density between groups also decreased when the districts were grouped according to the MoH and presidential definitions (remote/non-remote or less/more developed), as demonstrated by the reduced Theil-L between-group value for these definitions over time. However, the difference between groups increased when the districts were grouped according to the CBS definition (urban/rural). A similar situation was identified in China, where the gap in physician densities between the eastern, central and western regions has become more equitable over time, but the urban–rural gap in physician densities remains [32]. Findings in Indonesia and China suggest that which rural area definition is used could have an impact on how the unequal doctor distribution changes over time, which re-emphasizes the importance of choosing the fit-for-purpose rural area in policy monitoring and evaluation.

This study has several limitations. First, the literature collected was limited to key Indonesian government websites and peer-reviewed articles from selected databases. Hence, other rural area definitions not published online could have been overlooked. Second, the districts were grouped into those with and without remote health facilities to estimate the DPR and Theil-L indices for the MoH definition. Aggregating such information can cause a loss of granularity in data. However, sensitivity analyses grouping districts into quintiles according to the proportion of remote health facilities revealed a similar Theil-L decomposition and made no difference to the study conclusions. Third, while the inequality measures were discussed, this study does not explore the underlying causes of inequitable doctor distribution that may include, but are not limited to, population density, availability of public facilities, and distance to a capital or provincial capital [48–50]. Such inquiry is beyond the scope of this study but could be informative should policy-makers decide on key characteristics to be considered when defining rurality. Lastly, the empirical comparison of rural area classifications was based on DPRs. The validity of these definitions is likely different for other health policy and research fields that were not tested in this study. Thus, the findings are possibly quite specific for this health purpose. Further research is needed to validate whether these definitions are appropriate and optimal in other health policy areas. Until then, caution is recommended when generalizing to other areas of health policy or other sectors.

## Conclusion

Our study exemplifies the benefit of exploring and critically reviewing various rural area definitions in the light of developing a more fit-for-purpose definition for use in health policy and research. The Indonesian example revealed that the identified rural area definitions, while having different purposes, methods to categorize areas, and validity in measuring rurality, have some overlaps in identifying areas with low healthcare access. None of the definitions met all the ideal norms of urban/rural taxonomy. Strong collaboration between researchers and stakeholders is needed to help determine which characteristics should be investigated more, and which contextual aspects should be prioritized, when further developing rural area classifications to be used to inform rural health workforce policy.

This research identifies a range of considerations for informing fit-for-purpose country-level rural area definitions. First, there is a need to review the strengths and weaknesses of a country’s current or past rural area definitions which can be undertaken by using the proposed norms applied in this study. Second, objective measures like the Theil-L measure can be applied to national data such as DPRs to inform how accurate a definition is in differentiating rural areas where interventions should be targeted. Third, exploring factors associated with increasing rural doctor supply or capacity can fruitfully shape development of a rural area definition that is fit for health policy and research purposes. These could include spatial analyses, measuring incentive preferences among doctors, and investigating important local contexts. For example, considering local contexts in Indonesia, classifying rural areas at the district level would be aligned with decentralized governance, and hence, could optimize utility for policy-making.

## Supplementary Information

**Additional file 1. Table 1A** Selected policies with rural-area definitions that are related to health policy. **Table 2A** Rural area definitions identified. **Table 3A** Proportion of rural villages and less developed districts in 34 Provinces, 2018. **Table 4A** Scoring of Ministry of Health definition of remote health facilities. **Table 5A** Scoring of the Presidential-regulated definition of less-developed districts. **Table 6A** Scoring of Central Bureau of Statistics definition of urban-rural village. **Table 7A** Districts according to the Ministry of Health (MoH), Presidential, and Central Bureau of Statistics (CBS) rural area definitions.

## Data Availability

Data on doctors analysed in this study were obtained from the Central Bureau of Statistics. These data are not publicly available and were used under license for the current study. District population data are publicly available from the 34 websites of the provincial bureau of statistics.

## Abbreviations

CBS: Central Bureau of Statistics
GIS: Geographic information system
LMICs: Low- and middle-income countries
MoH: Ministry of Health
MoVDT: Ministry of Villages, Development of Disadvantaged Regions, and Transmigration
PODES: *Potensi Desa* (village census)
DPR: Doctor-to-population ratio
Puskesmas: *Pusat Kesehatan Masyarakat* (government-owned primary healthcare clinic)
