# Supplementary material for: A critical review of definitions of rural areas in Indonesia and implications for health workforce policy and research
Source: Health Res Policy Syst. 2022 Apr 27;20:46. doi: 10.1186/s12961-022-00847-w (PMC9044606; doi:10.1186/s12961-022-00847-w)
Supplement: Supplementary file 1 — Additional file 1. Table 1A Selected policies with rural-area definitions that are related to health policy. Table 2A Rural area definitions identified. Table 3A Proportion of rural villages and less developed districts in 34 Provinces, 2018. Table 4A Scoring of Ministry of Health definition of remote health facilities. Table 5A Scoring of the Presidential-regulated definition of less-developed districts. Table 6A Scoring of Central Bureau of Statistics definition of urban-rural village. Table 7A Districts according to the Ministry of Health (MoH), Presidential, and Central Bureau of Statistics (CBS) rural area definitions. [file 12961_2022_847_MOESM1_ESM.docx]

Additional file

Table 1A. Selected policies with rural-area definitions that are related to health policy

| **National** | |
| --- | --- |
| **Within the Ministry of Health (MoH)** | |
|  | MoH Regulation 949/MENKES/PER/VIII/2007 with criteria for remote and very remote health facilities |
|  | MoH Regulation 6/2013 with criteria for remote, very remote, and unattractive health facilities |
|  | Government Regulation 78/2014 on acceleration of development for less-developed areas |
|  | MoH Regulation 75/2014 on community healthcare centres |
|  | MoH Regulation 90/2015 on healthcare delivery in remote and very remote health facilities |
|  | MoH Decree HK.02.02/MENKES/110/2015 on the National Prioritised Program for 48 districts and 124 community health centres in border areas, 2015–2019 |
|  | MoH Regulation 52/2016 on the Standard Tariff of Health Service Provision in the National Health Insurance Program |
|  | MoH Regulation 10/2017 on Operational Instructions for the Use of the Special Allocation Fund for Health Infrastructure, fiscal year 2017 |
|  | MoH – Board for Development and Empowerment of Human Resources for Health Letter DG.01.01/II/1979/2018 on results of data verification for remote and very remote Puskesmas |
|  | MoH Regulation 43/2019 on community healthcare centres |
|  | MoH Decree HK.01.07/MENKES/545/2019 on financial incentives for the specialist deployment program |
| **External to the Ministry of Health** | |
|  | Presidential Regulation 7/2005 on the mid-term national development plan, 2004–2009 |
|  | Presidential Regulation 5/2010 on the mid-term national development plan, 2010–2014 |
|  | Head of Central Bureau of Statistics Regulation 37/2010 |
|  | Presidential Regulation 179/2014 on the spatial plan for country border areas in East Nusa Tenggara |
|  | Presidential Regulation 131/2015 on the determination of less-developed areas, 2015–2019 |
|  | Presidential Regulation 34/2015 on the spatial plan for country border areas in North Maluku and West Papua province |
|  | Minister of Villages, Development of Less-Developed Areas, and Transmigration Regulation 3/2016 on national technical guidance for determining indicators for less-developed areas |
|  | Ministry of Finance – List of LPDP Scholarship Affirmation Areas, 2019 |
|  | Presidential Regulation 63/2020 on determination of less-developed areas, 2020–2024 |
| **Local regulations** | |
|  | Mayor of Bandung Decree 440/Kep.657-DINKES/2012 on remote health facilities in Bandung City |
|  | Regent of North Luwu Decree 188.4.45/224/IV/2014 on categories of regular, remote and very remote community health centres, auxiliary community health centres, village health and maternity posts in North Luwu |
|  | Governor of Maluku Regulation 55/2017 on healthcare delivery in remote and very remote health facilities |
|  | Regent of North Tapanuli Regulation 13/2017 on financial incentives for government employees working in villages and remote and very remote health facilities in North Tapanuli |
|  | Regent of South Lampung Decree B/638/IV.03/HK/2019 on financial incentives for local officials, district health authority officials, staff at community health centres, internship doctors, specialist doctors, community empowerment for sanitation program consultants, and health personnel in remote areas in South Lampung, fiscal year 2019 |

Table 2A. Rural area definitions identified

| Classification | Inclusion criteria | |
| --- | --- | --- |
|  | Used in health-related policy/program | Clear scoring system |
| Meeting inclusion criteria | | |
| 1. Remote health facilities (MoH definition) | Doctor deployment for Nusantara Sehat or PTTD program, higher rate of capitation | Yes, 12 indicators |
| 1. Less-developed (underdeveloped) districts (Presidential definition) | Scholarship for specialist education | Yes, 27 indicators with composite index |
| 1. Urban-rural village classification (CBS definition) | Some of the criteria were used to classify Puskesmas' scope of work | Yes, 8 indicators |
| Not meeting inclusion criteria | | |
| 1. District nomenclature (*Kota / Kabupaten*) | No | Based on the name of each district, but no specific criteria. |
| 1. Underdeveloped, border, island areas (DTPK: *Daerah Tertinggal, Perbatasan, dan Kepulauan*) | Doctor deployment for *Nusantara Sehat* or PTTD program, higher incentives for specialist doctors, priority to receive health infrastructure funding | There is a regulation definition for 'underdeveloped' and 'border', but not for 'island'. Only 'underdeveloped' has clear scoring criteria as mentioned in point 2 in this table. |
| 1. Underdeveloped, frontiers, outermost areas (*Daerah* 3T: *Tertinggal, Terdepan, Terluar*) | This term is often used to determine areas prioritised to receive specialisation scholarships, and to employ doctors for the *Nusantara Sehat* or PTTD program. | There is a regulation definition for 'underdeveloped' and 'frontiers and outermost', but only 'underdeveloped' has clear scoring criteria as mentioned in point 2 in this table. |
| 1. Village nomenclature (*Desa* / *Kelurahan*) | No | Based on the name of each village. |
| 1. Regional (Sumatera, Java-Bali, Kalimantan, Sulawesi, Nusa Tenggara-Maluku-Papua) | Incentives for internships were determined according to this classification. | It is not clear why some regions are classified as 'vulnerable' or otherwise. |
| 1. Cities, low-density district, high-density district | No | Based on the nomenclature (*Kota / Kabupaten*) for each district combined with population density. |

Notes:

PTTD: *PTT Daerah,* voluntary rural incentives program sponsored by local government

Table 3A. Proportion of rural villages and less developed districts in 34 Provinces, 2018

| Province | Villages | Rural villages | | Districts | Less-developed districts | |
| --- | --- | --- | --- | --- | --- | --- |
|  | n | n | % | n | n | % |
| Aceh | 6,508 | 5,759 | 88.5 | 23 | 1 | 4.3 |
| Bali | 716 | 447 | 62.4 | 9 | 0 | 0.0 |
| Bangka Belitung Island | 391 | 263 | 67.3 | 7 | 0 | 0.0 |
| Banten | 1,552 | 979 | 63.1 | 8 | 2 | 25.0 |
| Bengkulu | 1,514 | 1,354 | 89.4 | 10 | 1 | 10.0 |
| Central Java | 8,559 | 5,863 | 68.5 | 35 | 0 | 0.0 |
| Central Kalimantan | 1,576 | 1,477 | 93.7 | 14 | 1 | 7.1 |
| Central Sulawesi | 2,020 | 1,876 | 92.9 | 13 | 9 | 69.2 |
| East Java | 8,496 | 5,674 | 66.8 | 38 | 4 | 10.5 |
| East Kalimantan | 1,038 | 840 | 80.9 | 10 | 1 | 10.0 |
| East Nusa Tenggara | 3,353 | 3,154 | 94.1 | 22 | 18 | 81.8 |
| Gorontalo | 734 | 599 | 81.6 | 6 | 3 | 50.0 |
| Jakarta Capital Region | 267 | 0 | 0.0 | 6 | 0 | 0.0 |
| Jambi | 1,562 | 1,375 | 88.0 | 11 | 0 | 0.0 |
| Lampung | 2,654 | 2,336 | 88.0 | 15 | 2 | 13.3 |
| Maluku | 1,240 | 1,138 | 91.8 | 11 | 8 | 72.7 |
| North Kalimantan | 482 | 444 | 92.1 | 5 | 1 | 20.0 |
| North Maluku | 1,196 | 1,084 | 90.6 | 10 | 6 | 60.0 |
| North Sulawesi | 1,838 | 1,471 | 80.0 | 15 | 0 | 0.0 |
| North Sumatera | 6,132 | 5,099 | 83.2 | 33 | 4 | 12.1 |
| Papua | 5,552 | 5,393 | 97.1 | 29 | 26 | 89.7 |
| Riau | 1,875 | 1,604 | 85.5 | 12 | 0 | 0.0 |
| Riau Island | 416 | 270 | 64.9 | 7 | 0 | 0.0 |
| South Kalimantan | 2,008 | 1,744 | 86.9 | 13 | 1 | 7.7 |
| South Sulawesi | 3,049 | 2,563 | 84.1 | 24 | 1 | 4.2 |
| South Sumatera | 3,262 | 2,890 | 88.6 | 17 | 2 | 11.8 |
| Southeast Sulawesi | 2,354 | 2,167 | 92.1 | 17 | 3 | 17.6 |
| West Sumatera | 1,275 | 913 | 71.6 | 19 | 3 | 15.8 |
| West Java | 5,957 | 3,285 | 55.1 | 27 | 0 | 0.0 |
| West Kalimantan | 2,137 | 1,982 | 92.7 | 14 | 8 | 57.1 |
| West Nusa Tenggara | 1,143 | 808 | 70.7 | 10 | 8 | 80.0 |
| West Papua | 1,987 | 1,904 | 95.8 | 13 | 7 | 53.8 |
| West Sulawesi | 650 | 600 | 92.3 | 6 | 2 | 33.3 |
| Yogyakarta Special Region | 438 | 247 | 56.4 | 5 | 0 | 0.0 |

Table 4A. Scoring of Ministry of Health definition of remote health facilities

| No. | Criteria | Maximum score |
| --- | --- | --- |
| 1 | Situated inland, on a mountain, or on the coast | 1 |
| 2 | Situated on a small island or groups of islands | 2 |
| 3 | Situated in disaster-prone areas such as those prone to volcanic eruption, earthquake, or landslide | 1 |
| 4 | Public transport (land/water/air) to the district centre is only available once a week | 2 |
| 5 | Duration of a return trip to the district centre using public transport (land/water) takes more than 6 hours | 2 |
| 6 | Existing transportation may be disrupted by the weather | 1 |
| 7 | Difficulty in supplying food-related staples | 1 |
| 8 | Unstable security condition | 2 |
|  | Maximum total score | 12 |
| Final scoring: score of ≥3=remote | | |

Source: Ministry of Health regulation 90/2015

Notes:

Health facilities with a score of 3 up to 6 are considered remote and more than 6 as very remote. *Puskesmas* that score less than 3 can still be classified as 'remote' if they: 1) have limited access to reach all population within the catchment area, 2) are located more than 100 km from the catchment area boundaries; or 3) they are isolated from their catchment areas by geographic features such as rivers, seas, mountains, valleys, or forests. The policy on capitation rate, hardship allowance, and placement for the rural incentive programs for the remote and very remote facilities are the same.

Table 5A. Scoring of the Presidential-regulated definition of less-developed districts

| Key criteria | Factor | Direction | Weight |
| --- | --- | --- | --- |
| Economic | Proportion of population living in poverty | Positive | 10% |
|  | Per capita consumption | Negative | 10% |
| Fiscal capacity | Indicators of local financial capacity | Negative | 10% |
| Human resources | Life expectancy | Negative | 10% |
|  | Average length of schooling | Negative | 5% |
|  | Literacy rate | Negative | 5% |
| Infrastructure | Proportion of villages with majority asphalt / concrete roads | Negative | 1.5% |
|  | Proportion of villages with majority gravel roads | Positive | 1.5% |
|  | Proportion of villages with majority dirt roads | Positive | 1.5% |
|  | Proportion of villages with other types of roads | Negative | 1.5% |
|  | Proportion of households with phone lines | Negative | 2% |
|  | Proportion of households with electricity | Negative | 2% |
|  | Proportion of households with safe water | Negative | 2% |
|  | Proportion of villages with non-permanent or semi-permanent building market | Positive | 2% |
|  | Health facilities per 1,000 population | Negative | 2% |
|  | Doctors per 1,000 population | Negative | 2% |
|  | Primary and junior secondary schools per 1,000 population | Negative | 2% |
| Accessibility | Average distance from village office to district office | Positive | 6.67% |
|  | Proportion of villages with distance to health facility >=5km | Positive | 6.67% |
|  | Average distance from village to basic education service centre | Positive | 6.67% |
| Local characteristics | Proportion of villages prone to earthquake | Positive | 1.43% |
|  | Proportion of villages prone to landslide | Positive | 1.43% |
|  | Proportion of villages prone to flood | Positive | 1.43% |
|  | Proportion of villages prone to other natural disasters | Positive | 1.43% |
|  | Proportion of villages located in forest conservation | Positive | 1.43% |
|  | Proportion of villages located in *restrictive land* | Positive | 1.43% |
|  | Proportion of villages prone to conflict in the last 1 year | Positive | 1.43% |
| Final composite index (CI):  CI_min_ and CI_max_ was calculated across all districts  The higher the CI, the less developed.  Interval (i) is the difference between (CI_min_ and CI_max_)/5  Less-developed: if CI_district_ is >= CI_min_+2i | | | |

Source: Ministry of Village, Development of Disadvantaged Regions, and Transmigration 3/2016 on Technical Guidance for Determining Indicators of Less-developed Regions.

Table 6A. Scoring of Central Bureau of Statistics definition of urban-rural village

| Characteristics | Criteria | Score |
| --- | --- | --- |
| Population density/km^2^ | <500 | 1 |
|  | 500 – 1249 | 2 |
|  | 1250 – 2499 | 3 |
|  | 2500 – 3999 | 4 |
|  | 4000 – 5999 | 5 |
|  | 6000 – 7499 | 6 |
|  | 7500 – 8499 | 7 |
|  | > 8500 | 8 |
| Proportion of farming households | > 70 | 1 |
|  | 50 – 69.9 | 2 |
|  | 30 – 49.9 | 3 |
|  | 20 – 29.9 | 4 |
|  | 15 – 19.9 | 5 |
|  | 10 – 14.9 | 6 |
|  | 5 – 9.9 | 7 |
|  | < 5 | 8 |
| Kindergarten or junior secondary school or senior secondary school | Available in the village, or distance between the village and facility =<2.5 km | 1 |
|  | Distance between village and facility >2.5 km | 0 |
| Traditional markets or shops or convenience store | Available in the village, or distance between the village and facility =<2 km | 1 |
|  | Distance between village and facility >2 km | 0 |
| Cinema or hospital | Available in the village, or distance between the village and facility =<5 km | 1 |
|  | Distance between village and facility >5 km | 0 |
| Hotel / night club / hairdresser | Available | 1 |
|  | Not available | 0 |
| Phone line | Proportion of households with phone line >=8% | 1 |
|  | Proportion of households with phone line <8% | 0 |
| Electricity | Proportion of households with electricity >=90% | 1 |
|  | Proportion of households with electricity <90% | 0 |
| Final scoring:  Total score: 2 – 22  10 – 22 = Urban village  <10 = Rural village | | |

Source: Head of Central Bureau of Statistics regulation 37/2010

Table 7A. Districts according to the Ministry of Health (MoH), Presidential, and Central Bureau of Statistics (CBS) rural area definitions

| **MoH definition^1)^** | **Presidential definition^2)^** | **CBS definition^3)^** |
| --- | --- | --- |
| Without remote health facilities  (n=271) | More developed  (n=235) | Q1, most urban (n=93) |
|  |  | Q2 (n=55) |
|  |  | Q3 (n=47) |
|  |  | Q4 (n=21) |
|  |  | Q5, most rural (n=19) |
|  | Less-developed  (n=36) | Q1, most urban (n=0) |
|  |  | Q2 (n=3) |
|  |  | Q3 (n=4) |
|  |  | Q4 (n=10) |
|  |  | Q5, most rural (n=19) |
| With remote health facilities  (n=243) | More developed  (n=157) | Q1, most urban (n=10) |
|  |  | Q2 (n=37) |
|  |  | Q3 (n=36) |
|  |  | Q4 (n=50) |
|  |  | Q5, most rural (n=24) |
|  | Less-developed  (n=86) | Q1, most urban (n=0) |
|  |  | Q2 (n=8) |
|  |  | Q3 (n=16) |
|  |  | Q4 (n=22) |
|  |  | Q5, most rural (n=40) |

Notes:

The table shows how districts were grouped for analyses in this paper.

1. For the MoH definition, we grouped districts according to the presence of at least one remote health facility in the district, as verified by Ministry of Health letter DG.01.01/II/1979/2018.
2. More- or less-developed districts are defined in Presidential Regulation 131/2015.
3. For the CBS definition, we grouped districts into 5 quintiles according to the proportion of the district population residing in rural villages. Dividing the districts based on quintiles was considered a better method than calculating the doctor-to-population ratio for groups of urban and rural villages separately for each district. The latter method resulted in under-or overestimation compared to the former (Additional File, Table 7A). The proportion of people residing in rural villages, divided into quintiles, was calculated from data in Central Bureau of Statistics Regulation 37/2010. The range of rural population percentages at each quintile was: 0–32.79 (Q1), 32.84–67.36 (Q2), 67.64–79.16 (Q3), 79.25–90.11 (Q4), and 90.15–100 (Q5).

Table 8A. Inequality measures of the doctor-to-population ratio (DPR) according to the urban-rural village classification

| Year | Theil-L total^3^ | Theil-L decomposition | |
| --- | --- | --- | --- |
|  |  | % Within group^4^ | % Between group^5^ |
| 2011 | 0.42 | 67 | 33 |
| 2014 | 0.44 | 60 | 40 |
| 2018 | 0.42 | 60 | 40 |

Source of data: Village Census 2011, 2014, and 2018, based on the number of doctors in the village.

Doctor-to-population ratios were calculated separately for urban and rural areas in each district.

1. Theil L total of doctor-to-population ratio in Indonesia
2. Decomposition of Theil L that reflects the difference of DPR within each group (L_W_)
3. Decomposition of Theil L that reflects the difference of DPR between groups (L_B_)
